# Marked paper: Type 2 diabetes mellitus indicates increased postoperative complications and poor prognosis in colorectal cancer patients receiving curative surgery

**DOI:** 10.3389/fonc.2023.1128383

**Published:** 2023-02-09

**Authors:** Daoli Liu, Xubing Zhang, Hong Zhou, Zhiqiang Zhu, Yiren He, Xiao Wan, Bo Zhang, Shaojun Liu, Liu Liu

**Affiliations:** ^1^ Department of Gastrointestinal Surgery, Anqing First People’s Hospital of Anhui Medical University, An Qing, An Hui, China; ^2^ Department of General Surgery, The First Affiliated Hospital of USTC, Division of Life Sciences and Medicine, University of Science and Technology of China, Hefei, Anhui, China; ^3^ Department of General Surgery, The Anhui Provincial Hospital affiliated to the An Hui Medical University, He Fei, An Hui, China

**Keywords:** diabetes mellitus, colorectal cancer, complication, prognosis, surgery

## Abstract

**Purpose:**

This study aimed to evaluate the impact of type 2 diabetes mellitus (T2DM) on the short-term outcomes and long-term survival of patients with colorectal cancer (CRC) who underwent curative resection.

**Methods:**

This study retrospectively included 136 patients (T2DM group) with resectable CRC and T2DM from Jan 2013 to Dec 2017. Propensity score-matched control group consisting of 136 patients (non-T2DM group) were selected from 1143 CRC patients without T2DM. The short-term outcomes and prognosis were compared between the T2DM and non-T2DM group.

**Results:**

A total of 272 patients (136 patients for each group) were included in this study. Patients in T2DM group had higher body mass index (BMI), higher proportion of hypertension and cerebrovascular diseases (P<0.05). T2DM group had more overall complications (P=0.001), more major complications (P=0.003) and higher risk of reoperation (P=0.007) when compared with non-T2DM patients. T2DM patients had longer hospitalization time than non-T2DM (20.7 ± 10.2 *vs*. 17.5 ± 6.2, P=0.002). As for the prognosis, T2DM patients had worse 5-year overall survival (OS) (P=0.024) and 5-year disease-free survival (DFS) (P=0.019) in all stage. Moreover, T2DM and TNM stage were the independent predictors of OS and DFS for CRC patients.

**Conclusions:**

T2DM increases overall complications and major complications, and prolongs the hospitalization time after CRC surgery. In addition, T2DM indicates the poor prognosis of CRC patients. A prospective study with large sample size is required to confirm our findings.

## Introduction

Colorectal cancer (CRC) is the third most common malignancy in China, with over 550,000 patients newly diagnosed in 2020, and the fifth most common cause for cancer-related deaths, with more than 280,000 deaths in 2020 (1, 2). Unlike European countries and northern American countries, the incidence and mortality of CRC continue to increase in China (2). Although the CRC treatment strategies, including chemotherapy, targeted therapy and Immunotherapy, develop rapidly, and the prognosis of CRC improves greatly, surgery remains the cornerstone in the treatment of CRC (3, 4).

Type 2 diabetes mellitus (T2DM) is highly prevalent over the past decades, and expected to be 700 million in 2045 (5). T2DM has been suggested to be associated with risks of many types of cancers, including liver cancer, gastric cancer, colorectal cancer and renal cancer (6–9). In addition, T2DM affects the prognosis of many types of cancers (10, 11). It has been reported that T2DM significantly increased Lymph node involvement, and median disease-free survival is 81 months for non-T2DM patients and 36 months for T2DM patients, with breast cancer (11). Similarly, patients with lung cancer and T2DM had significantly higher mortality (HR=1.27, 95% CI: 1.07-1.50) than non-T2DM patients, irrespectively receiving insulin or metformin treatment (12).

The association between T2DM and risk of CRC has been investigated in some studies, and most studies suggested a negative impact of T2DM on the incidence of CRC (13, 14). The impact of T2DM on the prognosis of CRC has been studied in many studies; however, these studies yielded inconsistent results (15–18). A recent meta-analysis suggested that T2DM led to poor survival and increased the risk of relapse in CRC patients (17). However, some studies suggested no effect of T2DM on the disease-specific and the all-cause survival among CRC patients (16). On the other hand, the impact of T2DM on the short-term outcomes and prognosis of CRC patients receiving curative surgery remained uncertain (15, 19). In the present study, we investigate the effect of T2DM on the short-term outcomes and prognosis of patients with CRC and T2DM who underwent curative surgery.

## Patients and methods

### Study population

Patients diagnosed with CRC who underwent primary curative surgery were retrospectively included in a single center between Jan 2013 and Dec 2017. All patients were histologically diagnosed with CRC adenocarcinoma preoperatively. A total of 136 CRC patients with T2DM were included as the T2DM group. A total of 1143 CRC patients without T2DM were screened, and 136 patients were selected as the non-T2DM group by matching gender with T2DM group using propensity score matching (20). Color Doppler echocardiography, abdominal-enhanced computed tomography (CT) and plain chest CT were routinely performed as a part of the preoperative assessment. Other inclusion criteria included the following (1): age older than 18 (2); underwent primary curative surgery for CRC; and (3) no severe cardiopulmonary dysfunction. The exclusion criteria include (1): emergency surgery for gastrointestinal massive bleeding, obstruction or perforation (2); stage IV CRC (3); Non-R_0_ resection; and (4) incomplete medical record. Informed consent was obtained from each patient. This study was conducted in accordance with the World Medical Association Declaration of Helsinki, and approved by the Ethics Committee of our hospital (No.2022-RE-116).

### Follow-up

All patients were monitored with CEA, CA199 and body CT scan every 3 months for the first 3 years, and every 6 months for the following 2 years. Colonoscopy was conducted every year after surgery. Survival status was verified using the review of out-patient records and/or telephone interviews with patients or patients’ family. The overall survival (OS) defined as the time from primary surgery to the last follow-up (when patients were alive) or death. Disease-free survival was defined as the time from primary surgery to the last follow-up, recurrence or death.

### Definitions

The tumor node metastasis (TNM) was diagnosed according to the AJCC eighth edition (21). The postoperative complications were defined according to the Clavien–Dindo classification, and major complications were defined as≥III classification complications (22). T2DM was diagnosed according to the American Diabetes Association Criteria (casual plasma glucose concentration≥200 mg/dL, or fasting plasma glucose≥126 mg/dL, or 2h glucose≥200 mg/dL after the Oral Glucose Tolerance Test) (23).

### Data collection

Clinical data of patients were retrospectively collected from patients’ medical records. The clinical data included age, gender, body mass index (BMI), hypertension, cardiovascular diseases, cerebrovascular diseases, American Society of Anesthesiologists (ASA) score, tumor location (colon or rectum), operative approach (open surgery or laparoscopic surgery), maximal tumor diameter, TNM stage, operative time, hospital stay, postoperative complications, anastomotic leakage and reoperation. There is no death in all patients. The prognosis included overall survival (OS) and disease-free survival (DFS).

### Statistical analysis

Continuous data are expressed as the mean ± standard deviation (SD), and Student’s t-test was used to compare the differences between the two groups. Categorical data are shown as n (%), and Chi-squared test or Fisher exact test were used. Continuous variables including age, BMI, the maximal tumor diameter and operative time were converted to categorical variables by the median values in all patients for the following logistic regression analyses and cox regression analyses. Univariate and multivariate logistic regression analyses were used to identify the factors associated with the overall complications. Survival rates were calculated with the Kaplan–Meier curve, and compared with log-rank tests. Multivariate cox regression analyses were conducted to identify independent predictive factors for OS and DFS. Data were analyzed using SPSS (version 22.0) statistical software. A bilateral p value <0.05 was considered statistically significant.

## Results

### Patient characteristics

A total of 136 CRC patients with T2DM (T2DM group) underwent primary curative surgery, and were included in study between Jan 2013 and Dec 2017. After propensity score matching for gender, 136 CRC patients without T2DM as a control group (non-T2DM group) were selected from 1143 CRC patients without T2DM. The characteristics of the T2DM and non-T2DM patients were shown in **Table 1**. The patient BMI in T2DM group was larger than patients in non-T2DM group (23.8 ± 3.5 kg/m^2^ *vs*. 22.7 ± 3.2 kg/m^2^, P=0.008). The proportion of patients with hypertension were higher in T2DM group than in non-T2DM group (57% *vs*. 29%, P<0.001). The rate of CRC patients with cerebrovascular diseases was also higher in in T2DM group than in non-T2DM group (14% *vs*. 4%, P=0.006).

**Table 1:** The basic characteristics of the included patients.

Variables	T2DM	Non-T2DM	P value
	n=136 (%)	n=136 (%)	
Gender			1.000
male	92 (73)	92 (73)	
female	44 (24)	44 (24)	
Age (years)	64.9 ± 11.1	63.2 ± 11.7	0.226
BMI(kg/m2)	23.8 ± 3.5	22.7 ± 3.2	0.008
Hypertension			<0.001
Yes	77 (57)	39 (29)	
No	59 (43)	97 (71)	
Cardiovascular diseases			0.303
Yes	10 (7)	6 (4)	
No	126 (93)	130 (96)	
Cerebrovascular diseases			0.006
Yes	19 (14)	6 (4)	
No	117 (86)	130 (96)	
ASA score			0.110
1&2	90 (66)	102 (75)	
3	46 (34)	34 (25)	
Tumor location			
Colon	64 (47)	56 (41)	
Rectum	72 (53)	80 (59)	
Operation approach			0.268
Open	61 (45)	52 (38)	
Laparoscopy	75 (55)	84 (62)	
Tumor diameter(cm)	4.3 ± 1.8	4.2 ± 1.7	0.957
TNM stage			0.194
I	15 (11)	23 (17)	
II	68 (50)	55 (40)	
III	53 (39)	58(43)	

### The impact of T2DM on the risk of postoperative complications

As shown in **Table 2**, T2DM group had significantly higher rate of overall complications than non-T2DM group (30% *vs*. 14%, P=0.002). In addition, T2DM group also had higher rate of major complication and re-operation than non-T2DM group (major complication risk: 7% *vs*. 0%, P=0.001; Re-operation risk: 6% *vs*. 0%, P=0.007). The detailed information for patients encountered major complications and re-operations was shown in **Supplementary table 1**. The time of hospital stay in T2DM group was significantly longer than that in non-T2DM group (20.7 ± 10.2 days *vs*. 17.5 ± 6.2, P=0.002). As shown in **Table 3**, Univariable logistic regression analyses showed that T2DM, hypertension, high ASA score and longer operative time predicted high risk of postoperative complications. Multivariable logistic regression analyses showed that T2DM and longer operative time were the independent predictors for postoperative complications.

**Table 2:** The short-term outcomes between the two groups.

Variables	T2DM	Non-T2DM	P value
	n=136 (%)	n=136 (%)	
Operative time	181.6 ± 61.1	176.9 ± 56.0	0.515
Hospital stay(days)	20.7 ± 10.2	17.5 ± 6.2	0.002
Surgical site infection	29 (21)	13 (10)	0.007
Anastomotic leakage	12 (9)	10 (7)	0.656
Major complications	9 (7)	0 (0)	0.003
Overall complications	41 (30)	19 (14)	0.001
Re-operation	8 (6)	0 (0)	0.007

**Table 3:** Univariate and multivariate analyses of overall complications.

Variables	Univariate analysis		Multivariate analysis	
	OR (95%CI)	P value	OR (95%CI)	P value
Gender (Female/male)	0.70 (0.37-1.32)	0.267		
Age (≥/< 60 years)	1.34 (0.72-2.47)	0.356		
BMI (≥/< 23.2 kg/m^2^)	0.58 (0.33-1.02)	0.056		
T2DM (Yes/No)	2.16 (1.19-3.91)	0.011	2.29 (1.21-4.36)	0.011
Hypertension (Yes/No)	1.88 (1.07-3.32)	0.029	1.60 (0.84-3.05)	0.154
Cardiovascular diseases (Yes/No)	0.81 (0.21-3.09)	0.759		
Cerebrovascular disease (Yes/No)	1.46 (0.65-3.28)	0.363		
ASA score (3/1&2)	1.84 (1.03-3.26)	0.038	1.75 (0.91-3.35)	0.091
Tumor location (Colon/Rectum)	1.27 (0.72-2.22)	0.408		
Operation approach (Laparoscopy/open)	1.08 (0.62-1.90)	0.778		
Tumor diameter (≥/< 4.3cm)	1.41 (0.80-2.47)	0.232		
Operative time (>/≤179min)	2.12 (1.19-3.18)	0.011	2.57 (1.39-4.74)	0.003
TNM stage (III stage/I&II stage)	0.77 (0.43-1.38)	0.378		

### The impact of T2DM on the prognosis of CRC

**Figure 1:**
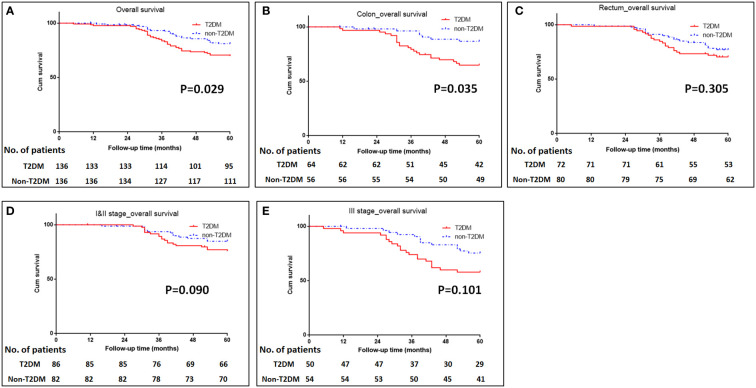
Kaplan-Meier analysis of survival in patients with T2DM. **(A)** Kaplan-Meier analysis of overall survival for all patients. **(B, C)** Kaplan-Meier analysis of overall survival for patients with colonic cancer **(B)** or rectal cancer **(C)**. **(D, E)** Kaplan-Meier analysis of overall survival for CRC patients with TNM I&II stage **(D)** or III stage **(E)**.

**Figure 2:**
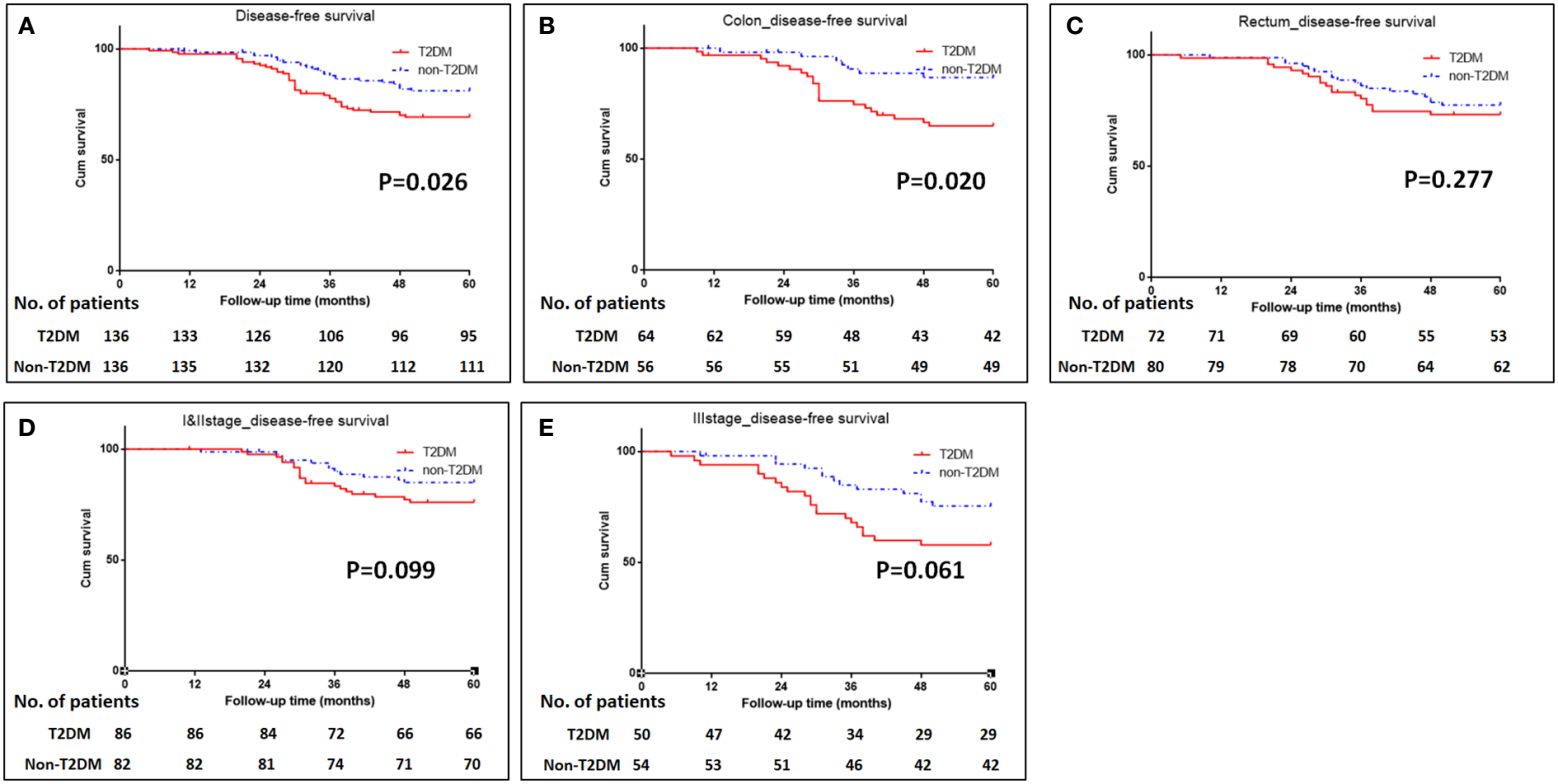
Kaplan-Meier analysis of disease-free survival in patients with T2DM. **(A)** Kaplan-Meier analysis of disease-free survival for all patients. **(B-C)** Kaplan-Meier analysis of disease-free survival for patients with colonic cancer **(B)** or rectal cancer **(C)**. **(D, E)** Kaplan-Meier analysis of disease-free survival for CRC patients with TNM I&II stage **(D)** or III stage **(E)**.

The median follow-up time for all patients was 68 (5–112) months. The 5-year OS were 70% for T2DM group and 82% for non-T2DM group. Log-rank test showed that 5-year OS rate was lower in T2DM group than in non-T2DM group (P=0.024) (**Table 4**) (**Figure 1A**). Multivariate cox regression analyses showed that T2DM and TNM-III stage remained the independent predictors for poor OS of CRC patients (HR (95%CI): 1.64 (1.01-2.65); P=0.46). The 5-year DFS were 69% for T2DM group and 81% for non-T2DM group. Similarly, 5-year DFS rate was lower in T2DM group than in non-T2DM group (P=0.026) (**Table 5**) (**Figure 2A**). Multivariate cox regression analyses showed that T2DM might independently predict poor DFS of CRC patients (HR (95%CI): 1.65 (1.00-2.73); P=0.05).

**Table 4:** Univariate and multivariate COX regression analyses of 5-year overall survival.

Variables	Univariate analysis		Multivariate analysis	
	HR (95%CI)	P value	HR (95%CI)	P value
Gender (Female/male)	0.99 (0.56-1.76)	0.986		
Age (≥/< 60 years)	1.14 (0.65-2.01)	0.652		
BMI (≥/< 23.2 kg/m^2^)	0.81 (0.47-1.39)	0.436		
T2DM (Yes/No)	1.83 (1.06-3.14)	0.029	1.64 (1.01-2.65)	0.046
Hypertension (Yes/No)	1.51 (0.88-2.58)	0.135		
Cardiovascular diseases (Yes/No)	1.35 (0.33-5.55)	0.675		
Cerebrovascular disease (Yes/No)	2.30 (0.99-5.32)	0.053		
ASA score (3/1&2)	1.12 (0.62-1.99)	0.712		
Tumor location (Colon/Rectum)	1.19 (0.70-2.04)	0.519		
Operation approach (Laparoscopy/open)	1.42 (0.83-2.43)	0.198		
Tumor diameter (≥/< 4.3cm)	1.02 (0.59-1.75)	0.943		
Operative time (>/≤179min)	1.33 (0.78-2.27)	0.297		
TNM stage (III stage/I&II stage)	1.77 (1.03-3.03)	0.038	1.78 (1.12-2.82)	0.015
No. of retrieved lymph nodes (≥/<12)	1.39 (0.69-2.82)	0.359		
Total complication (Yes/No)	1.96 (1.07-3.61)	0.030	1.40 (0.75-2.63)	0.295
Major complication (Yes/No)	3.51 (0.92-13.47)	0.067		
Anastomotic leakage (Yes/No)	2.42 (1.00-5.87)	0.050	1.58 (0.69-3.63)	0.283

**Table 5:** Univariate and multivariate COX regression analyses of 5-year disease-free survival.

Variables	Univariate analysis		Multivairate analysis	
	HR (95%CI)	P value	HR (95%CI)	P value
Gender (Female/male)	1.09 (0.61-1.96)	0.765		
Age (≥/< 60 years)	1.25 (0.69-2.25)	0.462		
BMI (≥/< 23.2 kg/m^2^)	0.71 (0.40-1.25)	0.230		
T2DM (Yes/No)	1.89 (1.08-3.31)	0.026	1.65 (1.00-2.73)	0.050
Hypertension (Yes/No)	1.08 (0.62-1.88)	0.777		
Cardiovascular diseases (Yes/No)	1.52 (0.37-6.26)	0.560		
Cerebrovascular disease (Yes/No)	1.79 (0.75-4.27)	0.187		
ASA score (3/1&2)	1.32 (0.73-2.37)	0.357		
Tumor location (Colon/Rectum)	1.08 (0.62-1.88)	0.778		
Operation approach (Laparoscopy/open)	1.43 (0.82-2.47)	0.207		
Tumor diameter (≥/< 4.3cm)	0.90 (0.52-1.58)	0.723		
Operative time (>/≤179min)	1.54 (0.89-2.68)	0.123		
TNM stage (III stage/I&II stage)	1.49 (0.86-2.59)	0.156		
No. of retrieved lymph nodes (≥/<12)	1.60 (0.75-3.38)	0.222		
Total complication (Yes/No)	1.25 (0.66-2.38)	0.500		
Major complication (Yes/No)	3.97 (1.03-15.23)	0.045	2.40 (0.94-6.11)	0.067
Anastomotic leakage (Yes/No)	1.81 (0.72-4.52)	0.204		

Subgroup analyses were conducted to investigate the impact of T2DM on the CRC prognosis with different TNM stages (TNM I&II or III stage) and on the different sites of CRC (colon or rectum). The results showed that patients with colon cancer and T2DM had poor OS and DFS than those with colon cancer and without T2DM (P=0.035 for OS, and 0.020 for DFS) (**Figures 1B, C**, **2B, C**). The impacts of T2DM on the prognosis of patients (including OS and DFS) with TNM I&II stage, or with TNM III stage or patients with rectal cancer did not reach statistically significances (**Figures 1D, E**, **2D, E**).

## Discussion

In present study, T2DM increased overall complications and major complications after CRC surgery. As for CRC prognosis, T2DM was an independent predictor for 5-year OS and 5-year DFS of CRC patients.

T2DM is highly prevalent in the world, and increases the global medical cost (5). In addition, T2DM increased the incidences of many types of malignancies (6, 8, 9), including colorectal cancer, pancreatic cancer, liver cancer and gastric cancer. The impact of T2DM on outcomes of resectal CRC remains unclear. Some studies reported that T2DM induced poor CRC OS (17–19), while other studies reported the null association between them (15, 24).

In this study, we found that the incidences of postoperative overall complications and major complications in our study patients were 22% and 3%, which was in agreement with previous reports (25–27). In addition, we found that CRC patients with T2DM had significantly higher overall complications and major complications than those without T2DM. Re-operation risk in T2DM group was also increased when compared with non-T2DM group. T2DM group had significantly longer time of hospitalization than those without T2DM, which was due to increased postoperative complications. In the multivariate analyses, T2DM and longer operative time were the independent risk factors for higher postoperative complications, which was in agreement with previous studies. In a retrospective study, Cheng et al. (15) showed that T2DM was significantly associated with significantly higher incidence of postoperative complications in CRC patients than those without T2DM (28.5% *vs*. 22.7%, P=0.033). Yap et al. reported T2DM was significantly associated with increased surgical complications (OR: 1.44, 95%CI: 1.02-2.04) and prolonged hospitalization time (linear regression coefficient 3.8 days, 95% CI 0.7–7.1) (28). Furthermore, a meta-analysis reported that T2DM significantly increased incidences of anastomotic leakage and surgical site infection (OR: 2.41, 95%CI: 1.84–3.16 for anastomotic leakage and OR: 1.98, 95%CI: 1.64–2.39 for surgical site infection) (29). The possible reasons are that T2DM leads to extensive microangiopathy, poor blood supply and thus poor tissue regeneration.

With regard to the long-term prognosis, the 5-year OS was 70% in T2DM group and 82% in non-T2DM group; the 5-year OS was significantly shorter in T2DM group than non-T2DM group (Hazard ratio: 1.83, 95%CI: 1.06-3.14, P=0.029). Multivariate cox regression analyses showed that T2DM and TNM III stage were the independent predictors for poor OS (adjusted Hazard ratio: 1.64, 95%CI: 1.01-2.65, P=0.046). Similarly, the 5-year DFS was 70% for T2DM group and 82% for non-T2DM group, and it was statistically significant between the T2DM and non-T2DM group (Hazard ratio: 1.89, 95%CI: 1.08-3.31, P=0.026). Multivariate cox regression analyses showed that T2DM marginally predicted poor DFS (adjusted Hazard ratio: 1.65, 95%CI: 1.00-2.73, P=0.05). Our findings were consistent with previous research results (19, 24, 30). Chen et al. reported that T2DM was the independent predictor for poor 5-year OS (adjusted Hazard ratio: 1.35, 95%CI: 1.20-1.51, P<0.001) (19). A meta-analysis included 26 studies demonstrated that CRC patients with T2DM were at greater risk for all-cause and cancer-specific mortality and have worse disease-free survival than those without T2DM (24). However, some studies reported null relationship between T2DM and CRC prognosis (15, 16, 18).

To further investigate the impact of T2DM on CRC in detail, subgroup analyses for CRC TNM stage (I&II stage and III stage) and for tumor locations (colon and rectum) showed that T2DM led to the poor survival of patients with colon cancer; however, the impact of T2DM on the prognosis of patients with different CRC TNM stage and rectal cancer did not reach statistical significance, which was probably due to the relatively small sample size. Hiroki Y et al. reported that patients with T2DM had 1.38-fold risk of colon cancer and 1.20-fold risk fo rectal cancer (31). Chen KH et al. found that patients with T2DM had significantly shorter 5-year overall survival (71% *vs*. 81.7% for T2DM *vs*. non-T2DM); after adjusting confunder factors of age, sex, stage, adjuvant chemotherapy, and comorbidities, T2DM remained the independent risk factor for 5-year overall survival (19). A comparative study conducted by Huang YC et al. confirmed that T2DM had significantly negative impact on 5-year overall survival in stage II colon cancer patients (HR: 1.21, 95% CI: 1.04-1.41, P=0.016) (32). These clinical data raised the hypothesis that T2DM probably had significantly negative impact on both colon cancer and rectal cancer, which should be assessed in the future study.

Although some hypotheses have been proposed, the mechanisms for the impact of T2DM on CRC prognosis remain unclear. First, T2DM could impact the poor prognosis of CRC through sharing the common risk factors, such as obesity and cerebrovascular diseases, which increased the mortality risk for CRC patients (33). Second, hyperinsulinemia has profound impact on the incidence and prognosis of CRC. Hyperinsulinemia can promote the cancer cell proliferation, chemo-resistance and metastasis through stimulating insulin and insulin-like growth factor-1 receptors (34, 35). Third, some studies showed that insulin-treated patients with T2DM had higher mortality from cancer than those without insulin treatment (19, 36). Metformin has been reported to reduce oxidative stress reaction and protect mitochondrial function; it can inhibit cancer cell proliferation and metastasis (37–39), and thus has a protective role in CRC prognosis. A meta-analysis showed that metformin might be a protective factor for CRC risk and prognosis in patients with T2DM (40).

There were some limitations in this study. First, this was a retrospective study, which encountered inherent bias that might affect the results of this study. Second, because the data regarding antidiabetic therapies were difficult to obtain, we did not analyze the impacts of antidiabetic drug on short-term outcomes and prognosis of CRC patients. Therefore, a prospective study with large sample size is required to provide more accurate results in the future.

## Conclusions

In conclusion, T2DM increases the incidence of postoperative complications and prolongs the hospitalization time after CRC surgery. In addition, T2DM independently predicts the poor survival of CRC patients. A prospective study is required to confirm our findings in the future.

## Data availability statement

The raw data supporting the conclusions of this article will be made available by the authors, without undue reservation.

## Ethics statement

The studies involving human participants were reviewed and approved by the Ethics Committee of our hospital (No.2022-RE-116). The patients/participants provided their written informed consent to participate in this study.

## Author contributions

Study concept and design: LL, DL, and SL. Collection and assembly of data: HZ, XZ, and BZ. Data analysis and interpretation: YH, XW, and ZZ. Manuscript writing: XW. Manuscript review and editing: ZZ and SL. All authors contributed to the article and approved the submitted version.
