# Supplementary material for: Marked paper: Type 2 diabetes mellitus indicates increased postoperative complications and poor prognosis in colorectal cancer patients receiving curative surgery
Source: Front Oncol. 2023 Feb 9;13:1128383. doi: 10.3389/fonc.2023.1128383 (PMC9947490; doi:10.3389/fonc.2023.1128383)
Supplement: Supplementary file 1 [file Table_1.docx]

**Supplementary table 1 The detailed information for patients with major complications and re-operation**

| **Patient No.** | **Group** | **Age (y); gender** | **Type of operation** | **Complication**  **/Re-operation** | **Treatment** | **Hospital stay (Days)** |
| --- | --- | --- | --- | --- | --- | --- |
| No. 1 | T2DM | 66; male | Abdominoperineal resection for rectal cancer | Wound dehiscence | Re-operation | 48 |
| No. 2 | T2DM | 66, female | Sigmoid resection for cancer | Anastomotic leakage | Re-operation | 38 |
| No. 3 | T2DM | 52, male | Dixon for rectal cancer | Anastomotic leakage | Re-operation | 41 |
| No. 4 | T2DM | 51, male | Laparoscopic Sigmoid resection for cancer | Anastomotic leakage | Re-operation | 17 |
| No. 5 | T2DM | 85, male | Laparoscopic right-side colon resection for cancer | Postoperative abdominal bleeding | Re-operation | 48 |
| No. 6 | T2DM | 51, male | Laparoscopic rectal resection for rectal cancer (Dixon procedure) | Postoperative abdominal bleeding | Re-operation | 43 |
| No. 7 | T2DM | 79, male | Laparoscopic rectal resection for rectal cancer (Dixon procedure) | Anastomotic leakage | Re-operation | 34 |
| No. 8 | T2DM | 88, female | Laparoscopic right-side colon resection for cancer | Abdominal infection | Conservative treatment | 41 |
| No. 9 | T2DM | 55, male | Laparoscopic right-side colon resection for cancer | Anastomotic leakage | Re-operation | 39 |
